# Patient and caregiver experience with delayed discharge from a hospital setting: A scoping review

**DOI:** 10.1111/hex.12916

**Published:** 2019-05-17

**Authors:** Amanda C. Everall, Sara J. T. Guilcher, Lauren Cadel, Maliha Asif, Joyce Li, Kerry Kuluski

**Affiliations:** ^1^ Leslie Dan Faculty of Pharmacy University of Toronto Toronto Ontario Canada; ^2^ Institute of Health Policy, Management & Evaluation University of Toronto Toronto Ontario Canada; ^3^ Lunenfeld‐Tanenbaum Research Institute Sinai Health System Toronto Ontario Canada

**Keywords:** burnout, caregivers, episode of care, live change events, patient care, patient discharge, patient preference, patient satisfaction, patient transfer, psychological

## Abstract

**Background:**

Delayed hospital discharge occurs when patients are medically cleared but remain hospitalized because a suitable care setting is not available. Delayed discharge typically results in reduced levels of treatment, placing patients at risk of functional decline, falls and hospital‐related adverse events. Caregivers often take on an active role in hospital to mitigate these risks.

**Objective:**

This scoping review aimed to summarize the literature on patient and caregiver experiences with delayed hospital discharge.

**Search strategy:**

Seven electronic databases and grey literature were searched using keywords including alternate level of care, delayed discharge, patients, caregivers and experiences.

**Inclusion criteria:**

Included articles met the following criteria: (a) patient or caregiver population 18 years or older; (b) delayed discharge from a hospital setting; (c) included experiences with delayed discharge; (d) peer‐reviewed or grey literature; and (e) published between 1 January 1998 and 16 July 2018.

**Data extraction:**

Data were extracted from the seven included articles using Microsoft Excel 2016 to facilitate a thorough analysis and comparison.

**Main results:**

Study themes were grouped into five elements of the delayed discharge experience: (1) overall uncertainty; (2) impact of hospital staff and physical environment; (3) mental and physical deterioration; (4) lack of engagement in decision making and need for advocacy; and (5) initial disbelief sometimes followed by reluctant acceptance.

**Conclusion:**

This review provides a foundation to guide future research, policies and practices to improve patient and caregiver experiences with delayed hospital discharge, including enhanced communication with patients and families and programmes to reduce deconditioning.

## 1 BACKGROUND

A common quality and safety concern in health systems across the developed world is patients’ inability to access needed services in a timely fashion. Delayed hospital discharge (also known as bed delay and, in Canada, alternate level of care) is one such quality concern, which occurs when a patient is medically cleared for discharge but remains hospitalized because a suitable care setting is not available.^1^, ^2^ In hospital, such patients often receive a significantly reduced level of treatment, rehabilitation and activation, placing them at risk of functional decline, falls and hospital‐related adverse events such as infectious disease and medication errors.^3^, ^4^, ^5^

Over the past several years, much attention has been paid to calculating the number of patients experiencing delayed hospital discharges and to understanding these patients' clinical characteristics and care destinations.^6^, ^7^, ^8^ Empirical studies have focused on identifying the sources, predictors and risk factors associated with delayed discharge including factors at the patient level, family and caregiver level, and organization and system levels.^6^, ^9^, ^10^, ^11^, ^12^, ^13^, ^14^ Briefly, patients experiencing delayed hospital discharge generally have complex health needs including physical and mental impairment.^6^, ^9^ Delayed hospital discharge has been associated with decreased abilities to participate in activities of daily living, frailty, increased age, high comorbidity (eg obesity and stroke), cognitive impairment, dependency and behavioural challenges.^9^, ^10^, ^11^, ^12^, ^14^, ^15^ One review has been published that included a brief summary of experiences of patients with delayed hospital discharge; however, the main focus of the review was on the impacts of delayed hospital discharge on patient health outcomes, evaluating associated costs and qualitatively assessing the impacts on patients, providers and hospitals.^15^

To address quality and safety concerns, health‐care delivery in the developed world is striving to become more patient‐ and family‐centred by capturing and addressing the needs and priorities of people and their families.^16^ Taking a person‐centred approach to care delivery is particularly important during care transitions when patients and caregivers are often at their most vulnerable.^16^, ^17^ Patient‐centred approaches are thought to improve patient health outcomes and experiences within the health‐care system.^16^ Unpaid caregivers, such as family members or friends, often take on an active caregiver role in hospital to mitigate the frequent reduction in clinical care.^18^, ^19^ Better understanding of the experiences of patients and caregivers could contribute to patient‐ and family‐centred approaches and strategies to address delayed hospital discharges.

The purpose of this scoping review was to summarize the scope of literature on the reported experiences of both patients and caregivers with delayed discharge from a hospital setting. Specifically, this scoping review focused on describing patient and caregiver perspectives towards delayed hospital discharge and the context surrounding delayed discharges (eg planned destinations, patient/caregiver characteristics), as well as identifying gaps and methodological approaches conducted to study this topic.

## 2 METHODS

A review protocol was created and amended in consultation with a librarian prior to the review commencing and is available from the researchers upon request. The protocol was not published or registered; however, the scoping review followed Levac's methodological framework and met the PRISMA‐ScR guidelines set out by Tricco and colleagues (Data S1).^20^, ^21^ The research question guiding this scoping review was: *What is known about the patient and unpaid caregiver experience with delayed discharge from a hospital setting*? The objectives were to identify: (a) the methodologies used to research this topic; (b) the study population characteristics (eg age, sex, socioeconomic status, comorbidities); (c) the definitions of delayed discharge guiding each study; (d) the experiences of patients and their caregivers with delayed hospital discharges; (e) the reasons for delayed discharges; and (f) the planned destination of patients who experienced delays.

Literature published in the past 20 years (between 1 January 1998 and 16 July 2018) was searched using the following seven electronic databases: MEDLINE (Ovid Interface), EMBASE (Ovid Interface), PsycINFO (Ovid Interface), Allied and Complementary Medicine Database (Ovid Interface), Cumulative Index to Nursing and Allied Health Literature (EBSCO Interface), Cochrane Library and Applied Social Sciences Index & Abstracts (ProQuest Interface). A 20‐year window was decided on due to the large number of records identified and the potential for older articles to be less relevant to today's health‐care systems. A search for grey literature was performed on TSpace, Canadian Institute of Health Information and the World Health Organization websites. The reference lists of included articles were also reviewed.

The search strategy was created in consultation with a librarian, and searches were conducted in each database using the appropriate Boolean operators, wild cards, proximity operators and truncations. A combination of the following keywords was searched: *alternate level of care, delayed discharge, bed blocking, bed occupancy, extended stay, patients, unpaid caregivers, experiences, perspectives, perceptions, satisfaction, expectation* and *attitude*. Synonyms for *unpaid caregivers* included *carer, family, friend, grandparent, mother, father, spouse, sibling* and *neighbour*. The initial search strategy used in MEDLINE was adapted for each additional database (see Data S1).

Articles from each of the seven databases were imported into the reference management software EndNote X8™. Duplicate articles were removed following Bramer's deduplication method by using custom import and export extensions to compare article citation information by changing display fields.^22^

The titles and abstracts of the 4725 articles were screened for the following inclusion criteria: (a) patient or caregiver population 18 years or older; (b) delayed discharge (ie medically cleared with no suitable next destination available) from a hospital setting; (c) included experiences with delayed discharge; (d) peer‐reviewed or grey literature; and (e) published between 1 January 1998 and 16 July 2018. Articles were excluded if they (a) were a book, book chapter, editorial, opinion piece, study protocol, case law or trial report, abstracts with no full‐text articles; (b) focused only on length of stay, impacts of delayed discharge on the hospital system or patient health outcomes (excluding experience); or (c) only described indicators/determinants of delayed discharge. Scoping and systematic reviews were also excluded; however, their reference lists were manually reviewed for relevant articles.

Titles and abstracts for the first 100 articles were screened independently by two individuals (AE and JL) using a Microsoft Excel (2016) spreadsheet with 98% agreement. Disagreements were discussed and resolved in an in‐person meeting between the two team members. Because a high level of agreement was achieved, the remaining articles were screened independently by one individual (AE), resulting in 59 articles remaining for full‐text review. Twelve of the 59 full‐text articles were reviewed independently by two individuals (AE and JL), and 100% agreement was obtained. The remaining articles were screened independently by one individual (AE), and seven articles were included in this scoping review.

Data were extracted from the seven included articles using Microsoft Excel 2016 to facilitate thorough analysis and comparison of the studies. Extracted data included general article information (eg publication date and country, authors and title), information on study characteristics (eg research question, study design and participant inclusion/exclusion criteria), patient and caregiver population characteristics (eg sample size, age, sex and ethnicity), pre‐ and post‐hospitalization details (eg event resulting in hospitalization, reason for delayed discharge and planned destination) and study outcomes, main findings and conclusions. Data were extracted only from the qualitative portions of the two mixed‐methods studies as the quantitative results did not address the research objective of this scoping review. Thematic analysis was used to synthesize the results from the included studies. This process involved multiple in‐person meetings between the research team until consensus was achieved. A critical appraisal of the included articles was not undertaken.

## 3 RESULTS

**Figure 1:**
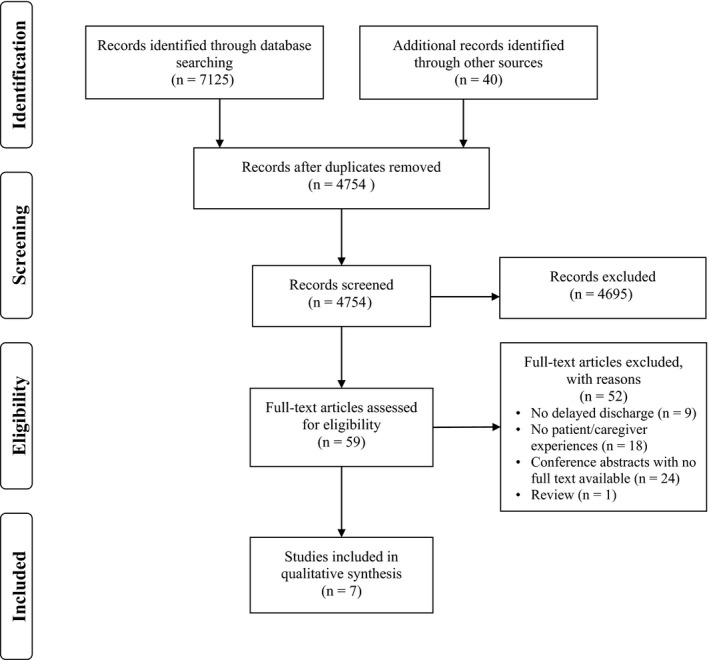
PRISMA flow diagram

The initial search resulted in 7125 articles and 40 additional records. After deduplication, 4754 articles remained for title and abstract screening (see Figure 1). Of the 59 full‐text articles that were reviewed, 52 were excluded. The remaining seven articles were included in this scoping review. The characteristics of the included articles are presented in Table 1. Of the seven included articles, five had qualitative study designs and two were mixed‐methods studies.^18^, ^19^, ^23^, ^24^, ^25^, ^26^, ^27^ Of the qualitative studies, the majority employed interviews (n = 4)^18^, ^19^, ^24^, ^26^ and one employed ethnography.^23^ Of the mixed‐methods studies, one employed a discussion based intervention with observational data collection^25^ and the other employed a combination of qualitative interviews, observations and a quantitative analysis of regional inpatient hospital data.^27^ The included studies were predominantly conducted in North America with four from Canada^18^, ^19^, ^24^, ^27^ and two from the United States.^25^, ^26^ The remaining study was conducted in the United Kingdom.^23^ All of the articles were published in the last 13 years.

**Table 1:** Characteristics of studies included in the scoping review (n = 7)

First Author (year) Country	Study objective	Study design	Sample characteristics (age, sex)	Total sample size
Cressman (2013),^18^ Canada	To describe older patients’ and family caregivers’ experiences with delayed discharge	Qualitative interviews	5 patients (82‐89 y; 3 females, 2 males) 4 caregivers (48‐59 y; 3 females, 1 male)	9
Kuluski (2017),^19^ Canada	To understand the experiences of family caregivers of patients experiencing a delayed discharge to a long‐term care facility	Qualitative interviews	15 caregivers (age not reported; 9 females, 6 males)	15^a^
Kydd (2008),^23^ United Kingdom	To describe frail, older patients’ lives after being classified as delayed discharge patients	Qualitative ethnography: interviews and observations	14 patients total (age and sex not reported) 3 patients in a detailed care report (age not reported; 3 females)	14^b^
McCloskey (2015),^24^ Canada	To provide insight into the experiences of patients with delayed discharge and their family members	Qualitative interviews	16 patients (mean age 85 y (SD 11.1); 11 females, 5 males) 4 caregivers (age not reported; 2 males, 1 female, 1 unknown)	20
Patrick (2006),^25^ United States	To assess the effectiveness of a group intervention designed to encourage discharges for patients hesitant to be discharged	Mixed‐methods study evaluating a group intervention	7 patients (age not reported; all male)	7
Swinkels (2009),^26^ United States	To assess older patients’ experiences with delayed discharge from an acute hospital setting	Qualitative interviews	23 patients (mean age 82 y (SD 5.4); 12 females, 11 males)	23
Wilson (2013),^27^ Canada	To understand older patients’ lived experiences as they waited in the hospital for discharge to a nursing home bed	Mixed methods: qualitative interviews, observations, photo‐voice; quantitative analysis of discharge data^c^	*Qualitative Phase*: 9 patients (ages 80‐92 y; 6 females, 3 males)	9

^a^ Fifteen family caregivers were interviewed pertaining to twelve individual patients.

^b^ Fourteen delayed discharge patients included in the study; however, detailed descriptions were provided for three patients only.

^c^ Only the qualitative portions of this study are reported in this scoping review.

Most articles (n = 5) provided a definition of delayed discharge or alternate level of care.^18^, ^19^, ^23^, ^24^, ^26^ Of the five studies that provided a definition for delayed discharge, four described patients as being either medically stable and cleared for discharge or no longer needing the intensity of service provided in their current setting.^18^, ^19^, ^24^, ^26^ The remaining article described delayed discharge as a situation ‘when a patient is inappropriately occupying a hospital bed’.^23^ Three of the five definitions attributed the delayed discharge to a lack of appropriate destination facilities or beds in such facilities.^19^, ^23^, ^26^

The median sample size of the qualitative portions of the seven included articles was 14 participants with a range of seven to 23 participants. One ethnographic study had a total sample size of 14 patients from which the study themes were derived; however, the article focused on case reports for three patients. Because the themes were derived based on data collected from all participants, the sample size used in the above calculation was 14.

Study participants were generally patients, with four studies including only patients,^23^, ^25^, ^26^, ^27^ two studies including patients and caregivers^18^, ^24^ and one study including only caregivers.^19^ The majority of articles included both male and female participants (n = 5). One article included only male participants^25^ and the ethnographic study^23^ contained three detailed case reports of three female participants. In the four articles that reported the age of patient participants, all were above 80 years old.^18^, ^24^, ^26^, ^27^ Four studies used a minimum age as inclusion criteria for selecting patient participants.^18^, ^23^, ^26^, ^27^ Of the three articles that included caregiver participants, only one reported the age of the caregiver,^18^ which ranged from 48 to 59 years old.^23^ Patient and caregiver marital statuses were both only reported in one article.^18^ Patient and caregiver ethnicity, income level and education level were not reported in any of the included articles.

In regard to health conditions of patients, four articles reported the primary condition or event resulting in patient hospitalization. These were most commonly mental illness or neurological/brain injuries (n = 11), falls (n = 9) or cardiovascular conditions (n = 4).^19^, ^23^, ^24^, ^25^ None of the included articles described patients’ secondary conditions or multimorbidity.

Of the seven included articles, three reported the living arrangements of the patient prior to hospitalization.^19^, ^23^, ^24^ Two articles described living arrangements as either living with family or a carer or living alone.^19^, ^24^ The third article was the ethnography, which described the patients as either living in a house or in community housing.^23^ The majority of the articles (n = 6) reported some information on the type of hospital in which participants were waiting; however, there was little consistency in the descriptions provided. All but one of the included articles described the planned destination for at least some of the patient following hospitalization.^18^, ^23^, ^24^, ^25^, ^26^, ^27^ These destinations were most frequently assisted‐living, long‐term care or nursing homes. Most studies (n = 5) reported the length of patients’ delayed discharge,^18^, ^19^, ^23^, ^25^, ^26^ which ranged from 11 days to over 6 years. One study explicitly reported the reason for delayed patient discharge stating that the patients did not wish to be discharged.^25^ Table 2 provides a summary of the delayed hospital discharges characteristics.

**Table 2:** Summary of delayed hospital discharge characteristics (n = 7)

Author (year)	Type of hospital(s) (n)	Event resulting in hospitalization (n)	Length of delayed discharge (n)	Post‐hospital destination	Experiences with delayed discharge
Cressman et al, (2013)^18^	Large urban teaching hospital (1)	Not reported (9)	11‐85 d (9)	LTC home Retirement home CCC facility	Patients and caregivers were uncertain about their future Patients had a pessimistic view of their future
Kuluski et al, (2017)^19^	Acute care and post‐acute care, rural hospitals (3)	Fall (5) Stroke (1) Cancer (1) Acquired brain injury (1) Mental illness (1) Frailty (2) Not reported (4)	<1 y (7) 1‐3 y (4) >3 y (1) Not reported (3)	Not reported	Caregivers were frustrated with wait times for placement in LTC homes Caregivers were dissatisfied with the lack of care provided to their loved ones during their extended hospital stay
Kydd, (2008)^23^	Not reported	Fall (1) Not reported (13)	3 mo (1) 8 mo (1) Not reported (12)	Rural care home LTC home	Patients and caregivers were uncertain about their future Patients were pessimistic of their future Patients’ had limited friendships with other patients because they were cognizant that their stays should be short Patients were affected by the mood of the health‐care providers and the staff caring for them
McCloskey et al, (2015)^24^	Mix of urban and rural and regional and community hospitals (3)	Dysphagia (1) Pain (1) UTI (1) Cardiac (3) Respiratory (2) Social admission (2) Fall (3) Neurological (2) Cellulitis (1) Not reported (4)	Not reported (20)	LTC facility Assisted care facility	Patients and caregivers became normalized to long wait times associated with delayed discharge Patients and caregivers were accepting (no frustration) of having to wait for care Some patients felt undeserving of clinician attention and felt they were a burden on the hospital staff
Patrick et al, (2006)^25^	Mental health hospital (1)	Schizophrenia or schizoaffective disorder (7)	6‐year mean (7)	Supported community housing Congregate community living programmes	Some participants were fearful of being denied placement Patients awaiting placement felt that the attempts to move them out of the hospital were futile
Swinkels and Mitchell, (2009)^26^	Acute care hospitals (3)	Not reported (23)	32‐d mean, ± 26 d (23)	Home Nursing home NHS Care	Patients were disillusioned with their extended hospital stay Patients were disengaged from discharge planning processes
Wilson et al, (2013)^27^	Full‐service hospitals (2)	Not reported (9)	72‐d mean (9)	Nursing home	Patients awaiting placement to another setting described the hospital environment as bleak

Abbreviations: CCC, complex continuing care facility; LTC, long‐term care; NHS, National Health Services.

### 3.1 Key themes of included studies

**Figure 2:**
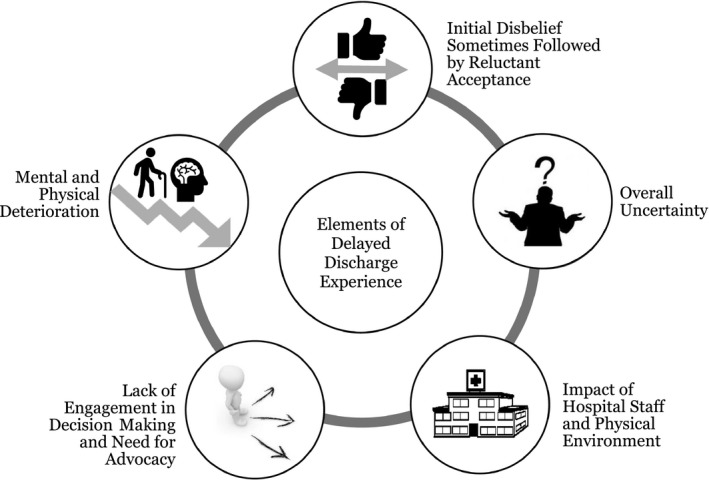
Elements of the delayed discharge experience

Authors of the included studies presented qualitative themes in six of the seven included articles. For the remaining article,^25^ our research team synthesized themes from the reported qualitative results. Study themes are presented in Table 3. Study themes from the included articles were grouped thematically into five overarching elements of the delayed discharge experience: (a) overall uncertainty; (b) impact of hospital staff and physical environment; (c) mental and physical deterioration; (d) lack of engagement in decision making and the need for advocacy; and (e) initial disbelief sometimes followed by reluctant acceptance of the situation (see Figure 2). Below is a description of each element of the delayed discharge experience.

**Table 3:** Themes identified from qualitative portions of studies (n = 7)

Author, (year)	Themes identified	Explanation of themes
Cressman et al, (2013)^18^	I never thought I'd end up like this	Most patients struggled to come to terms with their decline in functional ability and described their experiences being hospitalized with delayed discharge as discontinuous with their past experiences and preferences about their future
	I don't know	Patients described not knowing about the hospital processes, what questions to ask, the placement process after discharge, and their diagnosis and prognosis
	Waiting	Patients expressed a desire for more mobility, meaningful activity, care, placement and reunification with partners
Kuluski et al, (2017)^19^	Patient over person	Caregivers felt that the hospital environment caused clinicians to overlook patients’ non‐medical needs which led to the patients’ dignity and independence being compromised
	Uncertain and confusing process	Caregivers described feeling uncertain about clinicians’ decision making, length of waiting time, long‐term care destinations and when and how the placement would take place
	Inconsistent quality in care delivery	Caregivers expressed frustration with the lack of care, attention and time health‐care practitioners gave patients
	Carers addressing the gaps in the system	Despite having other responsibilities, caregivers provided patients with support when lacking and advocated for patients’ needs
	Personalization of long‐term care	Caregivers wanted patients be placed into a long‐term care facility with a private patient room that could be personalized according to patient preferences and was near the caregiver's home
Kydd, (2008)^23^	The effects of staff behaviour and attitudes upon the patients	Patients’ moods were influenced more by staff behaviours and attitudes than it was by the overall length of stay
	The patients’ experience	Patients were generally anxious about moving, were unaware of their diagnoses, avoided friendships with other patients (because they all knew they would be moving eventually)
	The environment and care	Staff used institutional rules to exercise power over patients, often favouring certain patients over others. Patients were aware that rules were used as a form of power and knew that some patients were favoured Boredom was the biggest complaint by patients, followed by having little choice in their future unless they had caregivers to advocate on their behalf
McCloskey et al, (2015)^24^	Perception of normalcy	Patients and caregivers had the perception that their pre‐hospitalization living conditions were normal despite experiencing difficulties like safety concerns, social isolation and dependency on others
	Old but not sick	Patients felt that they did not need acute hospital care and felt guilty about using hospital services and occupying a bed while waiting for long‐term care services
	Anticipating relocation to a long‐term care facility	Patients expressed wanting to leave the hospital and be expedited to a long‐term care setting, where they felt that they would have more autonomy, less social isolation and a better quality of life
Patrick et al, (2006)^a^, 25	The futility of ‘even trying to get out of here’	Some patients expressed anger and frustration towards the group intervention because they felt they were never going to leave the hospital
	A gradual transition over time towards accepting their discharge	Patients were initially quiet in the group intervention, but over months they became more engaged and some eventually accepted their discharge and transition out of hospital
Swinkels and Mitchell, (2009)^26^	The effects of delayed transfer	Patients were frustrated and experienced poor moods regarding the changes in their situations and reduction in mobility and were concerned about the effects of prolonged hospitalization on their health
	Involvement in planning for community discharge	Patients believed that they had no way of expediting their discharge from the hospital and felt that decisions about transfers to residential facilities were made by other individuals
	Community care needs	Most patients were unaware of the extent of their functional decline and underestimated the amount of community care they would need when they were discharged
Wilson et al, (2013)^27^	Coming to a realization of this significant move	*Subtheme (1) Realization and resignation*: All patients recognized that they would not be returning to their original place of residence because of their physical health decline *Subtheme (2) Decision‐making involvement*: Most patients described not being involved in the decision‐making process about transfer to a long‐term care facility, and some were upset about not being included in discussions about the care transfer
	Waiting is boring and distressing	*Subtheme (1) Waiting and more waiting*: Patients described feeling sad and frustrated about spending most of their day waiting in the hospital *Subtheme (2) Loneliness and social isolation*: Some patients described feeling lonely and socially isolated in the hospital, having very few visitors and little contact with the other patients in the ward
	Hospitals are not designed for waiting placement	*Subtheme (1) Few services or programmes*: There are very few programmes in place for patients that are awaiting placement (eg community events, group meals or bus trips) *Subtheme (2) Physical and mental stagnation*: A few patients described that their physical strength had deteriorated due to limited activities and being mostly bedbound. The interviewers observed that participants were not engaged in conversation and seemed to repeat themselves frequently

^a^ Themes derived by scoping review authors based on the reported qualitative results in the article.

#### 3.1.1 Overall uncertainty

Three qualitative studies described participants as uncertain about different aspects of their illness and treatment, hospital processes and their journey through the health‐care system.^18^, ^19^, ^23^ Cressman and colleagues, who conducted interviews with patients (n = 5) and caregivers (n = 4) in Ontario, Canada, found that the phenomenon of delayed discharge was characterized by uncertainty.^18^ More specifically, caregivers described feeling uncertain about what questions to ask and to whom to direct questions. Both patients and caregivers also felt uninformed about the results of medical assessments and diagnoses and described a lack of understanding of hospital and placement processes, which contributed to overall uncertainty. Kuluski and colleagues reported similar findings relating to uncertainty and confusion about hospital and transitional processes in their study involving interviews with 15 caregivers in Ontario, Canada.^19^ In this study, caregiver uncertainty extended to the duration of the delay, the final destination in which their family member (the patient) would be placed and how the placement would take place.

#### 3.1.2 Impact of hospital staff and physical environment

Three included articles reported that the hospital staff and/or the physical hospital environment impacted the overall patient and caregiver experience during delayed discharge.^19^, ^23^, ^27^ For example, an ethnographic study conducted by Kydd aimed to describe the lives of patients (n = 14) in the United Kingdom that experienced delayed discharge.^23^ The author of this study found that patients’ moods were directly affected by the attitudes and behaviours of hospital staff. Hospital staff were observed to exercise power over patients by selectively enforcing institutional rules for some patients, but not others. Patients were aware that rules were applied selectively and that ‘preferred’ patients were favoured over others, resulting in poor care experiences (ie feeling ‘angry, neglected or uncared for’).^23^ The physical environment of the hospital was also described as an inappropriate environment for delayed discharge patients.^19^, ^27^ The caregivers interviewed by Kuluski and colleagues expressed frustration at the lack of care, attention and time given to patients who were experiencing delays.^19^ Similarly, patients interviewed in the qualitative phase of Wilson and colleagues’ study described patients spending much of their day waiting, being socially isolated with few visitors with little contact with other hospital patients.^27^ Much of this isolation was described to be a result of the physical hospital environment and a lack of programmes to mentally and physically engage patients.

#### 3.1.3 Mental and physical deterioration

Four included articles described patients experiencing mental and physical deterioration during their delayed hospital discharge, often as a result of the lack of social and physical programmes and services in hospital.^18^, ^19^, ^26^, ^27^ Patients in two of the included studies expressed concerns about the effects of prolonged hospitalization on their overall health and a desire for more meaningful activities.^18^, ^26^ Specifically, patients voiced their concerns about reductions in mobility due to decreased activation and physical activity.^18^, ^26^ In Wilson and colleagues’ study, patients described deterioration of physical strength due to decreased activation and researchers observed patients to have limited social interactions.^27^ In Kuluski and colleagues’ study, caregivers echoed this concern and emphasized that patients’ non‐medical needs (eg social) were also important to ensure patients’ dignity and independence.^19^

#### 3.1.4 Lack of engagement and control in decision‐making processes and a need for advocacy

A lack of patient and caregiver involvement in the decision‐making process about transfers to other facilities was common among the included studies (n = 5).^19^, ^24^, ^25^, ^26^, ^27^ For example, Kuluski and colleagues found that a lack of engagement in the decision‐making process resulted in caregivers feeling that they had to advocate on behalf of the patient to ensure that his/her needs were being met in hospital and that patients were placed in an appropriate facility.^19^ Moreover, patients in Swinkels and Mitchell's study assessing patient experiences with delayed discharge from hospitals in the United States described feeling disempowered during the discharge planning process and felt they had little control over their situation, including how long the delay would take and decisions about their discharge destination.^26^ Patients in this study felt that the decision to transfer to nursing or residential homes was made by others and was associated with their deteriorating health and loss of independence.

#### 3.1.5 Initial disbelief sometimes followed by reluctant acceptance of the situation

Five articles reported themes related to an initial disbelief about the patients’ functional decline that resulted in the initial hospitalization and the delayed discharge situation.^18^, ^24^, ^25^, ^26^, ^27^ In two of these articles, this initial disbelief was followed by resignation or acceptance of the new circumstances.^25^, ^27^ In a Canadian study, McCloskey and colleagues interviewed patients (n = 16) and caregivers (n = 4) about their experiences with delayed hospital discharge and found that participants seemed to perceive the patients’ situation pre‐hospitalization as ‘normal’ even though many patients experienced safety issues, social isolation and dependency on others (eg friends, family, support workers). Some patients expressed feeling a sense of guilt over occupying a hospital bed while awaiting placement in a long‐term care home. Similarly, patients in three studies were described as either struggling to accept their situation or unaware of their decline in physical health and functional ability.^18^, ^26^, ^27^ Patrick and colleagues applied a mixed‐methods approach to assess an intervention that aimed at facilitating discharge of psychiatric patients (n = 7) who were hesitant to leave hospital in the United States.^25^ The intervention involved facilitated group sessions in which patients discussed their experiences in hospital and thoughts or goals of leaving the hospital. Researchers found that patients were initially quiet in the sessions; however, over time, patients generally became more engaged and accepting of their future discharge from hospital. Ultimately, five patients were discharged following the intervention.

### 3.2 Author recommendations

The authors of all of the included articles provided recommendations on how patients and caregivers could be better supported during delayed hospital discharges (Table 4). Recommendations included improvements at the interpersonal level, facility level and system level. Interpersonal‐level improvements included facilitating accurate and timely information sharing,^18^ assisting clinicians in engaging patients and caregivers in decision‐making processes^19^, ^23^, ^27^ and encouraging patients and caregivers to ask questions.^23^ Facility‐level improvements included developing guidelines and training staff on improving transitions,^23^, ^24^ creating policies to increase patient independence^25^ and increasing physical and mental activation of patients experiencing delays.^27^ System‐level improvements involved creating policies and processes, and advocating to decrease wait times for destination facilities^18^, ^24^, ^27^ and subsidizing funding for non‐hospital facilities.^24^

**Table 4:** Recommendations of Authors in Included Studies (n = 7)

Author (year)	Author recommendations
Cressman et al, (2013)^18^	Support patients through the delayed discharge experience Provide patients and families with timely and accurate information Promote patient recreation and mobility Revise policies to address LTC wait lists, reunifying couples and address copayments for delayed discharge
Kuluski et al, (2017)^19^	Equip clinicians with tools to help them engage with family caregivers Future studies should investigate the implications of formalizing the role of caregivers in the health‐care system
Kydd, (2008)^23^	Train staff on transitional states and care transitions Develop care plans with patients’ experiences in consideration Create avenues for patients to express questions and concerns Support staff and create avenues to share concerns during patient handovers
McCloskey et al, (2015)^24^	Develop strategies to facilitate the subsidization of home care Increase the number of long‐term care beds and develop a long‐term care wait‐list plan Develop guidelines for caring for patients experiencing delayed discharge
Patrick et al, (2006)^25^	Future research should: collect information about patient attitudes and behaviours on discharge using questionnaires and surveys use a control group to compare interventions to normal care
Swinkels and Mitchell, (2009)^26^	Restore patient independence through initiatives to improve the self‐esteem of patients experiencing delayed discharge
Wilson et al, (2013)^27^	Implement policies to involve patients awaiting placement in making decisions about their placement Take patients to visit the nursing homes where they will be placed to help with boredom and social isolation Provide patients with physical rehabilitation to prevent functional decline Increase advocacy for home care services for patients awaiting placement

## 4 DISCUSSION

A delayed hospital discharge is a critical care quality issue experienced by hospitals globally and much can be learned from the experiences of patients and their families on how to address the issue. Our scoping review found that few studies have captured patient and caregiver experiences on delayed discharge—particularly the caregiver experience. For example, during our search, only one systematic review was found that included patient or caregiver experiences with delayed hospital discharge. This review was conducted by Rojas‐Garcia and colleagues on the experiences of patients, health‐care providers and hospitals. They focused primarily on the impacts of delayed hospital discharge on patient health outcomes, evaluated associated costs and qualitatively assessed impacts on patients, providers and organizations.^15^ Their review included five studies on patient experience with delayed hospital discharge and provided a high‐level overview of the impact on patients: emotionally, patients felt worried and anxious about the delays, experiencing boredom; in regard to discharge planning, patients felt disengaged; and the lack of privacy and noise in hospital led patients to believe it was a poor environment for prolonged stays.^15^ While their high‐level summary includes some of our findings on patient experiences, their review did not include any studies on caregiver experiences.

The gap in research exploring caregiver experiences is critical to address, as patients with a delayed discharge are disproportionately impacted by cognitive impairments^4^ and may not be able to share their experiences. The seven studies that were captured in our scoping review point to gaps in two core areas in experiences with delayed discharge: (a) relational issues including communication and decision making and (b) lack of programmatic support during the delayed discharge period.

### 4.1 Relational issues

Relational issues are those relating to aspects of relationships (particularly, interactions between patients, caregivers and providers). The delayed hospital discharge period is a time of heightened vulnerability for patients and their caregivers; they are confused about what is currently happening to them, as well as what will happen next (including when the transition to the next care setting will take place).^18^, ^19^, ^23^ Open and ongoing dialogue with hospital staff is rare, and patient and caregiver feelings range from frustration to guilt (eg about occupying a bed).^19^, ^24^ Patients and caregivers want to be included in decision making but feel excluded from this process.^19^, ^27^ Feelings of powerlessness are common.^23^, ^24^, ^26^ The moods and lack of engagement of hospital staff also have an impact on the experiences of patients.^18^, ^19^, ^23^, ^26^, ^27^

### 4.2 Programmatic issues

Programmatic issues are those relating to programmes and services for patients. Overall, the scoping review findings showed that there was a lack of physical and mental health support during the delayed transition and experiences/concerns of physical deconditioning (ie physical deterioration) of patients. While hospital care was complete, patients still had care needs and there was confusion as to what these entitlements should be, and what the caregiver should be expected to do at this time.

In terms of moving the research and clinical agendas forward, much can be taken from the recommendations provided by the authors in the included studies, some which may be easier to implement in the shorter term, while others require greater shifts in the policy and care service landscapes (eg more care options such as assisted‐living and homecare). For example, Patrick and colleague's intervention could be easier to implement, at a hospital level, in the shorter term.^25^ They engaged patients with a delayed discharge in a mental health hospital who were reluctant to leave hospital (commonly cited as a barrier to discharge) in a series of focus groups. While this approach was initially resisted, patients opened up after a period of time. This demonstrates the need to take time to build relationships and increase comfort before real engagement can occur. Through the sharing of experiences and goal setting, this type of strategy provided an opportunity to reduce feelings of isolation while increasing involvement in future steps. This is a strategy that could be an important process change at the hospital level.

Clarity about roles and expectations for hospital staff, caregivers and patients is also needed. A communication strategy or process where patients and caregivers can continually connect with the care team (or designate) to ask questions and probe on next steps is required. Finally, a better understanding of the minimal service requirements for the delayed discharge period (which currently includes minimum to no services) is also needed, to avoid physical deterioration and reduce moral distress. While not the focus of this scoping review, the needs of managers and care providers also require consideration, including the tools and capacity required in their workdays to better engage patients with delayed discharge and caregivers.

#### 4.2.1 Limitations

There are a few limitations that should be noted. Firstly, it is possible that this scoping review missed relevant articles, as delay in discharge has several definitions, and 80% of full‐text articles were reviewed for inclusion by one independent reviewer. In order to minimize the possibility of missed articles, with the guidance of a senior librarian, our search strategy was adapted for a variety of databases and included all keywords and Mesh headings relating to delay in discharge and patient/caregiver experiences. Additionally, the reference lists of the included articles were manually searched for relevant articles. Secondly, a critical appraisal of the sources of evidence was not undertaken; however, this approach aligns with published method guidelines for scoping reviews.^21^, ^28^

#### 4.2.2 Future research

Our studies included mostly the patient perspective and that of older adults. Participant demographics (eg age, sex, ethnicity, income levels, educational levels, marital status, employment status, comorbidities) were generally poorly reported and should be captured more fully in future work so that differences in experience and needs by culture and language, social location, sex and gender can be better understood. Further, information about the types of hospitals and hospital units should be reported. Future research should explore, build on and test strategies to address the key concerns articulated in this paper, including engagement strategies, and continued services to reduce isolation and physical deconditioning. Importantly, to capture a fulsome understanding of the delayed discharge experience, experiences and barriers from the provider, manager and decision‐maker perspectives are also required in order to move towards implementable strategies to address delayed discharge challenges.

## 5 CONCLUSION

This review provides an important foundation to guide future research, policies and practices to improve patient and caregiver experiences with delayed hospital discharge, including enhanced communication with patients and families and programmes to reduce deconditioning.

## CONFLICT OF INTEREST

The authors report no conflicts of interest.

## Data Availability

Data sharing is not applicable to this article as no new data were created or analysed in this study.
