# Supplementary material for: Patient and caregiver experience with delayed discharge from a hospital setting: A scoping review
Source: Health Expect. 2019 May 17;22(5):863–73. doi: 10.1111/hex.12916 (PMC6803563; doi:10.1111/hex.12916)
Supplement: Supplementary file 1 [file HEX-22-863-s001.docx]

**SUPPLIMENTAL MATERIALS 2: DETAILED SEARCH STRATEGY**

**Title**: Patient and caregiver experience with delayed hospital discharge: A scoping review

MEDLINE: **Ovid MEDLINE: Epub Ahead of Print, In-Process & Other Non-Indexed Citations, Ovid MEDLINE® Daily and Ovid MEDLINE®**1946-Present

| **#** | **Search Term** | **Results** |
| --- | --- | --- |
| 1 | ((alternate level* adj2 care) or ALC).tw,kf. | 1936 |
| 2 | (bed adj2 (block* or occup* or delay*)).tw,kf. | 1098 |
| 3 | Bed Occupancy/ | 2429 |
| 4 | ((delay* or later or defer* or postpon*) adj2 (discharg* or transfer* or handoff* or handover* or releas*)).tw,kf. | 5034 |
| 5 | ((extend* or extension* or longer or lengthy or prolong*) adj2 stay*).tw,kf. | 11484 |
| 6 | **1 or 2 or 3 or 4 or 5** | **21496** |
| 7 | patients/ or inpatients/ | 36924 |
| 8 | (patient* or in?patient* or client*).tw,kf. | 6005942 |
| 9 | caregivers/ or family/ or nuclear family/ or grandparents/ or parents/ or mothers/ or fathers/ or spouses/ or siblings/ or friends/ | 208128 |
| 10 | (caregiver* or carer* or famil* or nuclear famil* or grandparent* or grandpa* or grandfather* or grandma* or grandmother* or parent* or mother* or father* or spouse* or sibling* or brother* or sister* or friend* or neighbo?r*).tw,kf. | 1650330 |
| 11 | **7 or 8 or 9 or 10** | **7301997** |
| 12 | exp patient satisfaction/ or attitude to health/ | 156803 |
| 13 | (experience or experiences or perspective or perspectives or percie* or perception or perceptions or satisfaction or expectation or expectations or attitude or attitudes or belie* or view*).tw,kf. | 1888116 |
| 14 | **12 or 13** | **1959594** |
| 15 | **6 and 11 and 14** | **2028** |
| 16 | **Limit 15 to yr=”1998-current”** | **1734** |

EMBASE: **Embase Classic+Embase**1947 to 2018 July 02

| **#** | **Search Term** | **Results** |
| --- | --- | --- |
| 1 | ((alternate level* adj2 care) or ALC).kw,tw. | 3548 |
| 2 | (bed adj2 (block* or occup* or delay*)).kw,tw. | 1792 |
| 3 | Hospital bed utilization/ | 3440 |
| 4 | ((delay* or later or defer* or postpon*) adj2 (discharg* or transfer* or handoff* or handover* or releas*)).kw,tw. | 8208 |
| 5 | ((extend* or extension* or longer or lengthy or prolong*) adj2 stay*).kw,tw. | 19000 |
| 6 | **1 or 2 or 3 or 4 or 5** | **34908** |
| 7 | Patient/ or Hospital patient/ | 1981615 |
| 8 | (patient* or in?patient* or client*).kw,tw. | 8974570 |
| 9 | caregiver/ or family/ or nuclear family/ or exp spouse/ or exp sibling/ or parent/ or father/ or mother/ or exp grandparent/ or friend/ | 401450 |
| 10 | (caregiver* or carer* or famil* or nuclear famil* or grandparent* or grandpa* or grandfather* or grandma* or grandmother* or parent* or mother* or father* or spouse* or husband* or wife* or sibling* or brother* or sister* or friend* or neighbo?r*).kw,tw. | 2123444 |
| 11 | **7 or 8 or 9 or 10** | **10524417** |
| 12 | Patient attitude/ or patient satisfaction/ or attitude to health/ | 273094 |
| 13 | (experience or experiences or perspective or perspectives or percie* or perception or perceptions or satisfaction or expectation or expectations or attitude or attitudes or belie* or view*).kw,tw | 2568142 |
| 14 | **12 or 13** | **2693950** |
| 15 | **6 and 11 and 14** | **3912** |
| 16 | **Limit 15 to yr=”1998-Current”** | **3511** |

PsychINFO: **PsycINFO**1806 to June Week 4 2018

| **#** | **Search Term** | **Results** |
| --- | --- | --- |
| 1 | ((alternate level* adj2 care) or ALC).tw,id. | 420 |
| 2 | (bed adj2 (block* or occup* or delay*)).tw,id. | 129 |
| 3 | ((delay* or later or defer* or postpon*) adj2 (discharg* or transfer* or handoff* or handover* or releas*)).tw,id. | 580 |
| 4 | ((extend* or extension* or longer or lengthy or prolong*) adj2 stay*).tw,id. | 1321 |
| 5 | **1 or 2 or 3 or 4** | **2439** |
| 6 | patient/ or hospitalized patient/ | 12215 |
| 7 | (patient* or in?patient* or client*).tw,id. | 768616 |
| 8 | caregivers/ or family/ or nuclear family/ or grandparents/ or parents/ or spouses/ or mothers/ or fathers/ or family members/ or siblings/ or brothers/ or sisters/ | 168210 |
| 9 | (caregiver* or carer* or famil* or nuclear famil* or grandparent* or grandpa* or grandfather* or grandma* or grandmother* or parent* or mother* or father* or spouse* or husband* or wife* or sibling* or brother* or sister* or friend* or neighbo?r*).tw,id | 733801 |
| 10 | **6 or 7 or 8 or 9** | **1388754** |
| 11 | health attitudes/ or "experiences (events)"/ or client attitudes/ or client satisfaction/ | 45993 |
| 12 | (experience or experiences or perspective or perspectives or percie* or perception or perceptions or satisfaction or expectation or expectations or attitude or attitudes or belie* or view*).tw,id. | 1436624 |
| 13 | **11 or 12** | **1444626** |
| 14 | **5 and 10 and 13** | **310** |
| 15 | **Limit 15 to yr=”1998 -current”** | **256** |

AMED: **AMED (Allied and Complementary Medicine)**1985 to June 2018

| # | **Search Term** | **Results** |
| --- | --- | --- |
| 1 | ((alternate level* adj2 care) or ALC).tw | 7 |
| 2 | (bed adj2 (block* or occup* or delay*)).tw | 17 |
| 3 | ((delay* or later or defer* or postpon*) adj2 (discharg* or transfer* or handoff* or handover* or releas*)).tw | 62 |
| 4 | ((extend* or extension* or longer or lengthy or prolong*) adj2 stay*).tw | 179 |
| 5 | **1 or 2 or 3 or 4** | **261** |
| 6 | patients/ or inpatients/ | 844 |
| 7 | (patient* or in?patient* or client*).tw. | 84070 |
| 8 | caregivers/ or family/ or exp parents/ or spouses/ | 6948 |
| 9 | (caregiver* or carer* or famil* or nuclear famil* or grandparent* or grandpa* or grandfather* or grandma* or grandmother* or parent* or mother* or father* or spouse* or sibling* or brother* or sister* or friend* or neighbo?r*).tw. | 21855 |
| 10 | **6 or 7 or 8 or 9** | **97139** |
| 11 | attitude/ or attitude to health/ or patient satisfaction/ |  |
| 12 | (experience or experiences or perspective or perspectives or percie* or perception or perceptions or satisfaction or expectation or expectations or attitude or attitudes or belie* or view*).tw. |  |
| 13 | **11 or 12** | **45440** |
| 14 | **5 and 10 and 13** | **41** |
| 15 | **limit 14 to yr="1998 -Current"** | **40** |

CINAHL: **CINAHL Plus with Full Text**

| **#** | **Search Term** | **Results** |
| --- | --- | --- |
| S16 | ( S6 AND S11 AND S14 ) AND DT 19980101-20180716 | **929** |
| S15 | S6 AND S11 AND S14 | **1005** |
| S14 | S12 OR S13 | **622913** |
| S13 | TI ( experience or experiences or perspective or perspectives or percie* or perception or perceptions or satisfaction or expectation or expectations or attitude or attitudes or belie* or view* ) OR AB ( experience or experiences or perspective or perspectives or percie* or perception or perceptions or satisfaction or expectation or expectations or attitude or attitudes or belie* or view* ) | 577781 |
| S12 | (MH "Patient Satisfaction") OR (MH "Attitude to Health") OR (MH "Attitude") | 92731 |
| S11 | **S7 OR S8 OR S9 OR S10** | **3224948** |
| S10 | TI (caregiver* or carer* or famil* or nuclear famil* or grandparent* or grandpa* or grandfather* or grandma* or grandmother* or parent* or mother* or father* or spouse* or sibling* or brother* or sister* or friend* or neighbo?r* ) OR AB ( caregiver* or carer* or famil* or nuclear famil* or grandparent* or grandpa* or grandfather* or grandma* or grandmother* or parent* or mother* or father* or spouse* or sibling* or brother* or sister* or friend* or neighbo?r* ) | 367446 |
| S9 | (MH "Grandparents") OR (MH "Family") OR (MH "Siblings") OR (MH "Spouses") OR (MH "Fathers") OR (MH "Parents") OR (MH "Mothers") OR (MH "Nuclear Family") OR (MH "Caregivers") | 125676 |
| S8 | TI ( (patient* or in#patient* or client*) ) OR AB ( (patient* or in#patient* or client*) ) | 3121400 |
| S7 | (MH "Inpatients") OR (MH "Patients") | 80924 |
| S6 | **S1 OR S2 OR S3 OR S4 OR S5** | **8003** |
| S5 | TI ( (extend* or extension* or longer or lengthy or prolong*) n1 stay* ) OR AB ( (extend* or extension* or longer or lengthy or prolong*) n1 stay* ) | 3250 |
| S4 | TI ( (delay* or later or defer* or postpon*) n1 (discharg* or transfer* or handoff* or handover* or releas*) ) OR AB ( (delay* or later or defer* or postpon*) n1 (discharg* or transfer* or handoff* or handover* or releas*) ) | 1014 |
| S3 | TI ( (bed n1 (block* or occup* or delay*)) ) OR AB ( (bed n1 (block* or occup* or delay*)) ) | 567 |
| S2 | (MH "Bed Occupancy") | 3196 |
| S1 | TI ( ((alternate level* n1 care) or ALC) ) OR AB ( ((alternate level* n1 care) or ALC) ) | 294 |

Cochrane

ID Search Hits

#1 ((alternate level* near/1 care) or ALC) ;ti,ab,kw 11

#2 (bed adj2 (block* or occup* or delay*)):ti,ab,kw 0

#3 [mh ^"bed occupancy"] 24

#4 ((delay* or later or defer* or postpon*) near/1 (discharg* or transfer* or handoff* or handover* or releas*)) .ti,ab,kw. 10

#5 ((extend* or extension* or longer or lengthy or prolong*) near/1 stay*) .ti,ab,kw. 16

#6 {or #1-#5} 61

#7 [mh ^patients] or [mh ^inpatients] 1382

#8 (patient* or in*patient* or client*) .ti,ab.kw 1209

#9 [mh ^caregiver] or [mh ^family] or [mh ^"nuclear family"] or [mh ^parents] or [mh ^fathers] or [mh ^mothers] or [mh ^spouses] or [mh ^siblings] or [mh ^grandparents] or [mh ^friends] 7692

#10 (caregiver* or carer* or famil* or nuclear famil* or grandparent* or grandpa* or grandfather* or grandma* or grandmother* or parent* or mother* or father* or spouse* or sibling* or brother* or sister* or friend* or neighbo*r*) .ti,ab,kw 742

#11 {or #7-#10} 10231

#12 [mh ^attitude] or [mh ^"attitudes to health"] or [mh ^"attitudes, health"] or [mh ^"patient satisfaction"] 15014

#13 (experience or experiences or perspective or perspectives or percie* or perception or perceptions or satisfaction or expectation or expectations or attitude or attitudes or belie* or view*) .ti,ab,kw. 1058

#14 #12 or #13 16065

#15 #6 and #11 and #14 34

#16 #6 and #11 and #14 Publication Year from 1998 to 2018 34

**Applied Social Sciences Index & Abstracts (ASSIA)**

(TI ((alternate level* NEAR/1 care) OR ALC) OR AB ((alternate level* NEAR/1 care) OR ALC)) OR (TI(bed NEAR/1 (block* OR occup* OR delay*)) OR AB(bed NEAR/1 (block* OR occup* OR delay*))) OR MAINSUBJECT.EXACT("bed occupancy") OR (TI ((delay* OR later OR defer* OR postpon*) NEAR/1 (discharg* OR transfer* OR handoff* OR handover* OR releas*)) OR AB((delay* OR later OR defer* OR postpon*) NEAR/1 (discharg* OR transfer* OR handoff* OR handover* OR releas*))) OR (TI((extend* OR extension* OR longer OR lengthy OR prolong*) NEAR/1 stay*) OR AB((extend* OR extension* OR longer OR lengthy OR prolong*) NEAR/1 stay*)) AND (MAINSUBJECT.EXACT(patients) OR MAINSUBJECT.EXACT(inpatients) OR MAINSUBJECT.EXACT(caregivers) OR MAINSUBJECT.EXACT(family) OR MAINSUBJECT.EXACT("nuclear family") OR MAINSUBJECT.EXACT(grandparents) OR MAINSUBJECT.EXACT(parents) OR MAINSUBJECT.EXACT(mothers) OR MAINSUBJECT.EXACT(fathers) OR MAINSUBJECT.EXACT(spouses) OR MAINSUBJECT.EXACT(siblings) OR MAINSUBJECT.EXACT(friends)) OR (TI (patient* OR in*patient* OR client* OR caregiver* OR carer* OR famil* OR nuclear famil* OR grandparent* OR grandpa* OR grandfather* OR grandma* OR grandmother* OR parent* OR mother* OR father* OR spouse* OR sibling* OR brother* OR sister* OR friend* OR neighbo*r*) OR AB(patient* OR in*patient* OR client* OR caregiver* OR carer* OR famil* OR nuclear famil* OR grandparent* OR grandpa* OR grandfather* OR grandma* OR grandmother* OR parent* OR mother* OR father* OR spouse* OR sibling* OR brother* OR sister* OR friend* OR neighbo*r*)) AND (MAINSUBJECT.EXACT.EXPLODE("Patient satisfaction") OR MAINSUBJECT.EXACT("attitude to health")) OR (TI (experience OR experiences OR perspective OR perspectives OR percie* OR perception OR perceptions OR satisfaction OR expectation OR expectations OR attitude OR attitudes OR belie* OR view*) OR AB(experience OR experiences OR perspective OR perspectives OR percie* OR perception OR perceptions OR satisfaction OR expectation OR expectations OR attitude OR attitudes OR belie* OR view*))

*Peer Reviewed and Additional limits – Date: After January 01 1998*

**Total 627**
